# Weak but Critical Links between Primary Somatosensory Centers and Motor Cortex during Movement

**DOI:** 10.3389/fnhum.2018.00001

**Published:** 2018-01-17

**Authors:** Pengxu Wei, Ruixue Bao, Zeping Lv, Bin Jing

**Affiliations:** ^1^Beijing Key Laboratory of Rehabilitation Technical Aids for Old-Age Disability, Key Laboratory of Neuro-functional Information and Rehabilitation Engineering of the Ministry of Civil Affairs, National Research Center for Rehabilitation Technical Aids, Beijing, China; ^2^Beijing Boai Hospital, School of Rehabilitation Medicine, China Rehabilitation Research Center, Capital Medical University, Beijing, China; ^3^School of Biomedical Engineering, Capital Medical University, Beijing, China

**Keywords:** motor control, functional magnetic resonance imaging, functional connectivity, weighted brain network, graph theory

## Abstract

Motor performance is improved by stimulation of the agonist muscle during movement. However, related brain mechanisms remain unknown. In this work, we perform a functional magnetic resonance imaging (fMRI) study in 21 healthy subjects under three different conditions: (1) movement of right ankle alone; (2) movement and simultaneous stimulation of the agonist muscle; or (3) movement and simultaneous stimulation of a control area. We constructed weighted brain networks for each condition by using functional connectivity. Network features were analyzed using graph theoretical approaches. We found that: (1) the second condition evokes the strongest and most widespread brain activations (5147 vs. 4419 and 2320 activated voxels); and (2) this condition also induces a unique network layout and changes hubs and the modular structure of the brain motor network by activating the most “silent” links between primary somatosensory centers and the motor cortex, particularly weak links from the thalamus to the left primary motor cortex (M1). Significant statistical differences were found when the strength values of the right cerebellum (*P* < 0.001) or the left thalamus (*P* = 0.006) were compared among the three conditions. Over the years, studies reported a small number of projections from the thalamus to the motor cortex. This is the first work to present functions of these pathways. These findings reveal mechanisms for enhancing motor function with somatosensory stimulation, and suggest that network function cannot be thoroughly understood when weak ties are disregarded.

## Introduction

Motor performance is improved by enhancing somatosensory inputs during movement. In sports training, athletic performance is improved by warm-up activities that include muscle contractions other than stretching (Fradkin et al., 2010). High-load dynamic warm-up programs improve the power and strength performance of athletes (McCrary et al., 2015); more intense warm-up trainings generate stronger sensory afferents and result in improved performance and power (Needham et al., 2009). In clinical applications, somatosensory stimulation promotes the motor function of patients suffering from motor deficits; this type of stimulation combined with voluntary muscle contractions induces more significant muscular adaptations than muscle contraction alone (Paillard, 2008; Kattenstroth et al., 2012). In addition, augmenting visual feedback with vibrotactile feedback is found to help subjects for reducing errors during motor learning (Bark et al., 2015).

All these findings feature a notable increase in somatosensory inputs during (Gulick et al., 2011) or very shortly before (Fradkin et al., 2010) voluntary movement. Limb movement excites afferent proprioception receptors in muscles and other soft tissues (Gardner and Johnson, 2013). Stimulating contracting muscles directly increases sensory inputs during movement (Gulick et al., 2011). Therefore, high-load dynamic warm-up training can more effectively activate these receptors than static forms, such as stretching.

Several studies further reported that when applied over the agonist muscle, somatosensory stimulation exhibits improved effects. In healthy people, electrical stimulation over the agonist muscle during exercise results in improved training effects than either electrical stimulation or exercise only (Willoughby and Simpson, 1998). In patients with knee-injury, voluntary exercise alone and voluntary exercise combined with non-simultaneous neuromuscular electrical stimulation only induce similar effects (Lepley et al., 2015), whereas muscle strength reduction can be better prevented by Quadriceps muscle contractions combined with simultaneous electrical stimulation compared with muscle contractions alone (Wigerstad-Lossing et al., 1988).

Mechanisms through which concurrent somatosensory stimulation changes the motor behavior during movement have not been reported yet in literature. Long-term electrical stimulation or motor training induces muscle adaptations (Paillard, 2008), whereas immediate changes in behavior mainly originate from the nervous system (Needham et al., 2009). Thus far, limited information is available regarding the immediate effects of stimulation during voluntary movement on the brain.

Muscle contraction is modulated by motor cortex in complex ways (Fujiwara and Rothwell, 2004; Gentner et al., 2008; Huang et al., 2008; Iezzi et al., 2008; Todd et al., 2009; Massie et al., 2013, 2015; Fang et al., 2014; Mirdamadi et al., 2015, 2017). A study using transcranial magnetic stimulation (TMS) found that somatosensory electrical stimulation strongly increased excitability of the primary motor cortex (M1; Veldman et al., 2014). Stimulation to the median or other nerves can modulate effects of TMS on motor cortex (Tokimura et al., 2000; Stefan et al., 2002), and dorsal root inputs very shortly prior to TMS can facilitate motor response (Roy et al., 2014). These findings indicate that modulating primary afferent connections to motor neurons can possibly change brain function.

Tactile-pressure stimulation shares some similarities with non-painful electrical stimuli applied in the aforementioned reports. Physical deformation of the tissue, such as pressure on the skin or stretch of muscles, is sensed by mechanoreceptors. Mechanoreceptors for touch and proprioception are innervated by dorsal root ganglion neurons with large-diameter, myelinated axons. These axons conduct action potentials rapidly and are activated by the non-painful electrical stimuli. In addition, such rapid conduction can provide the prompt sensory feedback required for motor control (Gardner and Johnson, 2013).

Aside from identifying the neural structures that constitute a functional brain system, characterizing the network properties that comprise functional interactions among many brain regions in the brain system is also important (Büchel et al., 1999). All brain regions involved in the tasks constitute a network. In a graph-theoretical analysis, the network can be defined as a graph, which is weighted when links (connections among brain regions) are assigned with weights. Weight value can be determined using the magnitude of temporal correlation between each pair of nodes (representing brain regions in the network; Rubinov and Sporns, 2010).

We hypothesize that during voluntary movement, simultaneous stimulation of the agonist muscle induces unique brain activation patterns and network properties compared with movement alone or movement combined with concurrent stimulation over areas without agonist muscles. In the present work, we perform functional magnetic resonance imaging (fMRI) to examine the activation and connectivity of the brain under three different conditions: (1) right ankle dorsiflexion (Task) as the baseline; (2) ankle dorsiflexion coupled with simultaneous stimulation to the agonist muscle (Task+AgonistStim); and (3) to a control area without agonist or antagonist muscles going through (Task+ControlStim).

## Materials and Methods

### Subjects

We recruited 22 right-handed healthy male volunteers aged 20–40 years (25.77 ± 6.27 years). Handedness was determined by a modified version of Annett’s Hand Preference Questionnaire and Edinburgh Handedness Inventory (Li and The National Cooperative Research Team for Handedness, 1983). One subject could not tolerate MR scanning due to the narrow space inside the scanner. Thus, data were acquired from 21 subjects. This study was carried out in accordance with the recommendations of National Research Center for Rehabilitation Technical Aids with written informed consent from all subjects. All subjects gave written informed consent in accordance with the Declaration of Helsinki. The protocol was approved by the ethics committee of National Research Center for Rehabilitation Technical Aids.

### MRI Scanning

Each volunteer was subjected to MR scanning with a Siemens Trio Tim 3T MRI system. Gradient echo images with blood-oxygen-level-dependent contrast were collected (TR = 3000 ms; TE = 40 ms; flip angle = 90°; field of view (FOV) = 240 mm × 240 mm; matrix size = 64 × 64). Samples consisted of thirty 5 mm-thick contiguous axial slices. T1-weighted images (3D MPRAGE sequence, TR = 1600 ms; TE = 2.15 ms; flip angle = 9°; inversion time = 800 ms; FOV = 256 mm × 256 mm; matrix size = 256 × 256) were also acquired.

### Motor Task

The duration of the fMRI experiment was 570 s for each subject. The fMRI session was composed of nine rest–task cycles with 30 s for each period. Eyes were kept closed during scanning. The motor task consisted of repetitive alternating dorsiflexion and relaxation of the right foot (with range reaching 15°). Foot movements were paced following an audio cue that was sounded every 1.5 s. These small range of motion and medium speed were applied to avoid large head motions. In each task period, the last audio cue is a verbal command “stop”. The verbal command, “ready, right foot movement, go” was delivered within a 2 s duration that is 3 s ahead of each task period. These audio cues and commands were recorded in advance and transmitted via the intercom system of the MR scanner. Each subject was trained prior to MR scanning to perform the motor task as gently as possible.

### Somatosensory Stimulation

Tactile-pressure stimulation was applied in six of the nine task periods. Three periods were allotted for stimulation of an area over the tibialis anterior (the agonist muscle of movement) or a control area proximal to the right medial malleolus (Supplementary Figure S1). Supplementary Figure S2 shows the sequence of the three conditions, namely, Task, Task+AgonistStim and Task+ControlStim.

A probe and a sponge were used to deliver tactile and pressure stimulations in human studies (Gracely et al., 2004; Stiasny-Kolster et al., 2004; Eickhoff et al., 2008). We modified the stimulation paradigm used in these studies. Tactile-pressure stimulation was applied using a wooden probe (length = 15 cm; diameter = 10 mm) covered with a sponge on the bottom end. Small lead blocks were attached onto the probe 5–8 cm from the bottom end to produce a 150 g mass load. The probe was tilted medially 45° relative to the vertical position when stimulating the control area and tilted laterally 45° when stimulating the tibialis anterior (Supplementary Figure S1).

Stimulation was administered along the long axis of the lower leg by brushing the skin of subjects in a back-and-forth manner with the probe at a frequency of 1 Hz and a shift distance of 3 cm. Compared with the smooth surface of a wooden probe, the sponge generated stronger tactile stimulation, resulting in stronger somatosensory input. When brushing the skin, different degrees of smoothness/coarseness of materials evoke different effects (Rolls et al., 2003).

### Activation Analysis

Image analysis was performed using SPM8 (Wellcome Department of Imaging Neuroscience, University College London, UK). Functional images were motion-corrected, co-registered with structural MR images, spatially normalized into the Montreal Neurological Institute (MNI) space, resampled to a voxel size of 3.0 mm × 3.0 mm × 3.0 mm and spatially smoothed using 6 mm full-width at half-maximum Gaussian kernel. Statistical analysis was performed at two levels by using the general linear model. Each condition (task vs. rest) was modeled using a boxcar function convolved with the hemodynamic response function. Significant changes in the signal intensity of each condition were identified using the mixed-effects model in group analysis. The threshold was set at a family-wise error rate correction of *P* < 0.05 with a minimum cluster extent of 10 contiguous voxels. Locations of brain activation, including peak *T* values, were defined using SPM Anatomy Toolbox (Eickhoff et al., 2005).

### Network Analysis

Functional connectivity is defined as the temporal correlations among spatially remote neurophysiological events. We constructed brain networks for each condition by using functional connectivity measured by task fMRI (Jbabdi et al., 2015). In this work, a brain area was selected as node when activated under the three conditions. Node centers (Supplementary Table S1) were determined by referring to literature data (Francis et al., 2009; Amiez and Petrides, 2014) or the center of mass of anatomical areas provided by SPM Anatomy Toolbox; the radius is 3 mm, similar to that used in a previous study (Wei et al., 2017). The waveform of every brain voxel was filtered using a bandpass filter (0.008 < *f* < 0.09) to reduce the effects of low-frequency drift and high-frequency noise. Six parameters obtained by rigid body head motion correction (three-rotation and three-translation parameters) were used as first-level covariates. Signals from ventricular regions, white matter, and their temporal derivatives were removed through linear regression. Pearson correlation coefficients among time courses of nodes were estimated with Conn toolbox designed for resting state and block data (Whitfield-Gabrieli et al., 2009). Correlation coefficients were converted into normally distributed scores by using Fisher’s transform for second-level random effect analysis.

Interactions between nodes in a network were presented as a weighted undirected graph, whose weights were represented by the Fisher-transformed correlation coefficient (beta value) for the corresponding link among network nodes. The threshold of the magnitude of correlations was *P* < 0.05, false discovery rate (FDR) corrected for multiple comparisons. The magnitude of correlations was used as link weights upon passing such threshold. No link was found between two nodes if the correlation failed to pass the threshold. Three networks corresponding to three conditions were separately constructed, and each network consisted of 23 nodes.

Network features (Supplementary Table S2) were analyzed using graph theoretical approaches with Brain Connectivity Toolbox. We measured distance *D* between two nodes with *D* = 1 − *W*, similar to that in a previous study (Achard and Bullmore, 2007), where *W* is the weight of the link between two nodes.

Node strength and betweenness are two measures for assessment of hubs in a network (Rubinov and Sporns, 2010). Betweenness of a node is the fraction of all shortest paths in networks passing through the node. This parameter is important in controlling information communication across separate parts of the network. In a binary network, the shortest path between two nodes is the path with the minimum number of links between a pair of nodes. Conversely, in a weighted network, the shortest path is the path between two nodes with the minimum sum of weights of constituent links. An optimal pathway is provided by the shortest path between two nodes because rapid transfer is achieved, and the use of system resources is minimized (Boccaletti et al., 2006).

Modules are groups of densely interconnected brain regions that are only sparsely connected to the rest of the network. Thus, brain regions within a module achieve a relatively fast rate of information transmission; different modules perform different functions with some degrees of independence (Bassett et al., 2011).

The obtained modules were compared with degree-, weight- and strength-preserving null models containing the same number of nodes (Rubinov and Sporns, 2011; Zalesky et al., 2012). For each network, we generated 100 degree-, weight- and strength-preserving null models containing the same number of nodes. The modular structure was subsequently identified for each constructed null model. Finally, we acquired 100 modular structures from the 100 constructed null models of each network. For each network, correlation coefficient values between the original modular structure and the 100 corresponding results were calculated. If low values were acquired, the modular structures acquired from the original networks are suggested to be unassociated with the weight, degree and strength properties of the original networks (Rubinov and Sporns, 2011).

Networks were visualized with BrainNet Viewer (Xia et al., 2013). Each network structure was also displayed using a force-spring algorithm (Kamada and Kawai, 1989) with Pajek (Batagelj and Mrvar, 1998) to obtain good network visualization (Hidalgo et al., 2007; Hagmann et al., 2008). The algorithm assumes that links are springs between all pairs of nodes and achieves the minimum total spring energy of networks. Two nodes show a larger distance when their connecting links have a lower summation value of weights.

## Results

We examined brain activations under three different conditions. All three conditions activated 23 brain regions, including the bilateral supplementary motor cortex (SMA), bilateral cingulate motor area (CMA), bilateral dorsal and ventral premotor cortex (PMd and PMv), bilateral secondary somatosensory cortex (S2), bilateral inferior parietal cortex (IPC), bilateral putamen, bilateral insula, bilateral cerebellum, left M1, left primary somatosensory cortex (S1), left superior parietal lobule (SPL), left thalamus and right middle frontal gyrus (MFG).

A weighted functional brain network of each condition is then constructed, and network properties are compared using graph theoretical analysis.

### Task+AgonistStim Evokes Most Extensive Brain Activations

**Figure 1:**
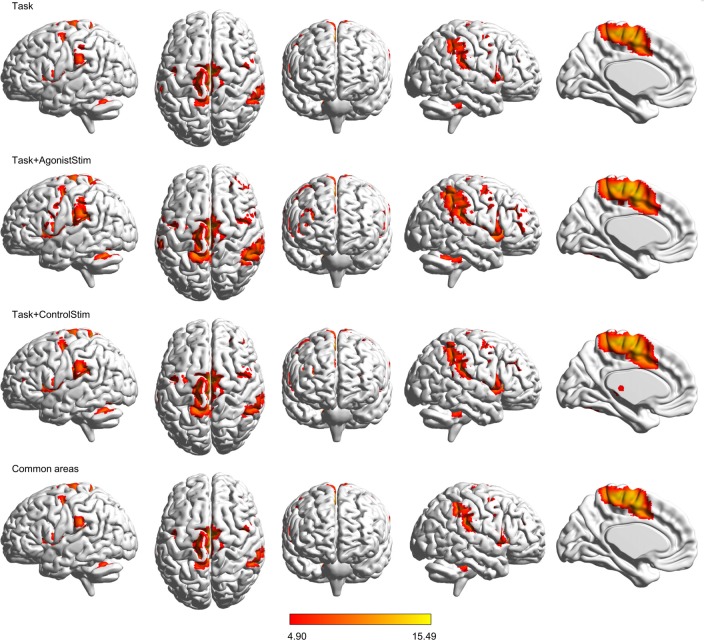
Brain activations. Activations evoked by different conditions are projected onto the normalized 3D brain with the BrainNet Viewer (http://www.nitrc.org/projects/bnv/). The threshold was set at a family-wise error rate correction of *P* < 0.05 with a minimum cluster extent of 10 contiguous voxels. The bottom row shows commonly activated areas shared by all three conditions; the last column is the midsagittal view of the left hemisphere. The colored bar indicates *t* values. Task+AgonistStim evokes the most extensive brain activations. Top to bottom rows consist of 2370, 5147, 4419 and 2320 activated voxels, respectively.

Task+AgonistStim and Task+ControlStim evoked more extensive brain activations than Task alone, with Task+AgonistStim evoking the most extensive brain activations (Figure 1). In addition, most peaks of Task+AgonistStim showed the highest *T* values over those of the two other conditions (Table 1). An activation cluster (containing 26 voxels from M1, S1 and SPL) was detected for the contrast “Task+AgonistStim minus Task” at a threshold *P* < 0.005 with 10 voxel extent, whereas no activation was found for any other pairwise comparisons.

**Table 1:** Peak activations.

Brain areas	Task				Task+AgonistStim				Task+ControlStim			
	MNI coordinates			*T* value	MNI coordinates			*T* value	MNI coordinates			*T* value
L_SMA	−6	−4	55	14.69	−6	−4	55	15.49	−6	−4	55	15.39
R_SMA	6	−4	61	11.15	9	5	61	12.58	6	−1	61	12.13
L_M1	−3	−28	64	13.75	−3	−28	64	15.18	−3	−28	64	14.85
L_CMA	−12	−7	49	8.90	−6	−7	49	12.22	−6	−7	49	11.73
R_CMA	12	2	45	7.82	3	8	46	10.33	9	14	34	8.37
L_PMd	−39	−10	55	6.52	−39	−10	52	7.89	−39	−10	52	7.50
R_PMd	45	−1	52	6.45	45	−1	52	7.93	45	−1	52	7.34
L_PMv	−54	5	7	6.43	−54	5	7	7.76	−48	5	4	7.72
R_PMv	57	8	16	7.70	57	8	16	9.12	57	8	16	7.79
R_MFG	36	41	25	5.92	36	41	25	7.57	36	41	25	7.47
L_S1	−12	−43	67	6.98	−12	−43	61	9.47	−12	−43	61	9.21
L_SPL	−12	−43	64	7.47	−6	−40	58	8.00	−12	−46	70	8.51
L_IPC	−63	−28	31	6.80	−63	−28	31	8.88	−60	−31	43	5.71
R_IPC	57	−28	25	9.05	57	−28	25	12.60	63	−28	22	11.29
L_S2	−48	−28	22	6.26	−48	−28	19	9.18	−48	−28	22	8.97
R_S2	54	−28	25	8.61	54	−28	25	12.43	54	−28	25	11.93
L_Insula	−45	2	8	6.63	−33	−25	19	7.35	−45	2	4	7.32
R_Insula	33	17	10	6.44	42	5	7	8.92	33	14	10	8.46
L_Putamen	−27	−1	13	7.78	−27	−1	13	9.81	−27	−1	13	9.01
R_Putamen	24	−1	13	5.44	24	−1	13	6.41	24	−1	13	7.03
L_Thalamus	−15	−13	4	6.41	−15	−13	4	6.85	−15	−13	4	7.31
L_Cerebellum	−33	−58	−26	7.40	−33	−58	−26	8.80	−33	−64	−26	9.20
R_Cerebellum	21	−34	−26	8.32	21	−34	−26	9.45	21	−34	−26	9.96

*Among all three conditions, activations evoked by Task+AgonistStim showed highest T values except L_SPL, R_Putamen, L_Thalamus, L_Cerebellum and R_Cerebellum*.

### Most Brain Regions Are Closely Connected Except for Primary Somatosensory Centers

Nodes for network construction include activated brain areas under all three conditions. The three networks contain only positive weighted links. Fully connected network of 23 nodes contains 253 links; Task, Task+AgonistStim, and Task+ControlStim networks comprise 176, 167 and 168 links, respectively.

**Figure 2:**
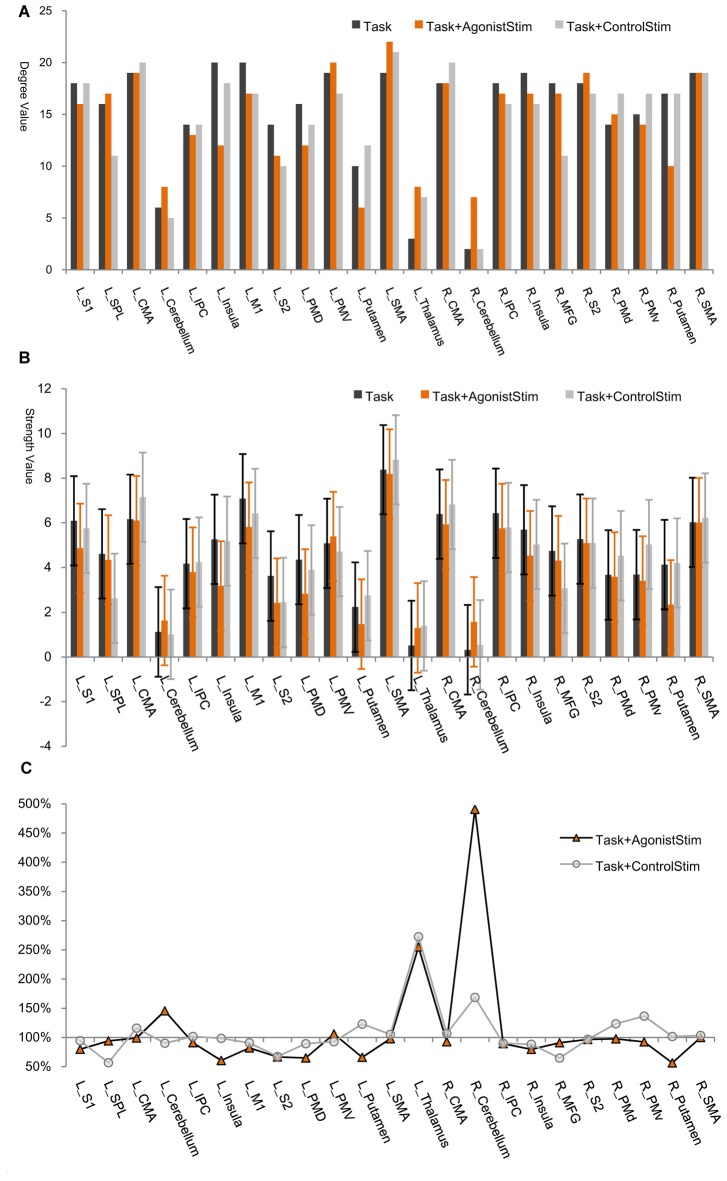
Node degree/strength values. **(A)** Degree values. The degree of a node is the number of links connected to this node. The thalamus, right cerebellum and left cerebellum exhibit the lowest degree values in all networks (only exception is the left putamen in the Task+AgonistStim network). R, right; L, left; SMA, supplementary motor area; M1, primary motor cortex; CMA, cingulate motor area; PMd, dorsal premotor cortex; PMv, ventral premotor cortex; MFG, middle frontal gyrus; S1, primary somatosensory area; SPL, superior parietal lobule; IPC, inferior parietal cortex; S2, secondary somatosensory area. **(B)** Strength values. Node strength is the sum of weights of all links connected to this node. The thalamus, right cerebellum and left cerebellum show the lowest strength values in all networks (except for L_Putamen in the Task+AgonistStim network). Error bars show ±SD. **(C)** Percentage changes in strength values. We use the strength value of each node in the Task network as the baseline (100%). The thalamus, right cerebellum and left cerebellum are nodes with overt increases in strength values in the Task+AgonistStim network, especially the right cerebellum. The Task+ControlStim network shows a distinctive pattern.

In each network, most nodes are highly connected to other nodes, and only a few (thalamus, right cerebellum and left cerebellum) incorporate small number of links (Figure 2). All these nodes are corresponding to primary somatosensory centers in the brain, i.e., the first places to receive somatosensory afferents from the peripheral nervous system via the spinal cord.

The thalamus, right cerebellum and left cerebellum are nodes with the lowest node strength values for all networks (Figure 2B). Node strength is the sum of weights of all links connected to a node and thus measures the extent of information transmission between this node and other nodes in a network. The three identified regions in the Task+AgonistStim network present increased strength values compared with those in the Task network. These increases were not replicated in the Task+ControlStim network. Comparison of the percentage change in strength values shows that the thalamus, right cerebellum and left cerebellum are nodes with overt increases in strength values in the network of Task+AgonistStim. However, a different trend was found in the Task+ControlStim network, i.e., lower levels of changes in right and left cerebellum but a higher level of changes in the thalamus when compared with the Task+AgonistStim network (Figure 2C).

### Task+AgonistStim Induces Unique Network Layout

**Figure 3:**
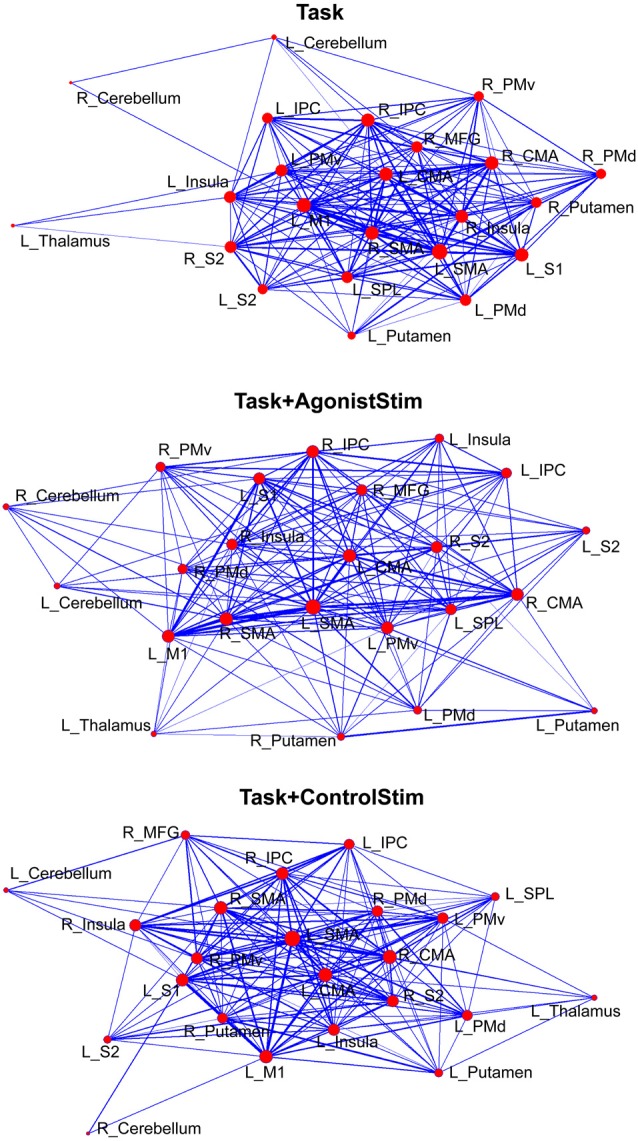
Different layouts of networks. Layouts are generated using the Kamada–Kawai force-spring layout algorithm (http://pajek.imfm.si). Nodes/links are sized according to their strength values or connection weights. This visualization method shows how brain regions are organized in a network. Each network shows a large “core” composed of densely-linked nodes with some sparsely connected peripheral nodes.

The network structure was displayed using a force-spring algorithm (Kamada and Kawai, 1989; Figure 3). Each network showed a closely connected “core”, which contained most cortical motor centers, such as SMA, M1 and CMA, and some peripheral nodes. In the Task network, the thalamus, right cerebellum and left cerebellum were nodes located far from the core; this finding is consistent with the lowest strength values of these nodes (Figure 2) and reveals that these primary somatosensory regions only play minor roles in the Task network. The Task+ControlStim network showed a similar pattern to the Task network.

The Task+AgonistStim network exhibited a pattern close to a homogenous distribution; the core part of this network was sparser, whereas the peripheral part was denser than the Task network. The thalamus, right cerebellum and left cerebellum were not the outermost nodes in the network.

### Network Hubs of Task+AgonistStim Differ from Those of the Other Conditions

L_SMA and L_M1 show the highest strength values in the Task network; meanwhile, many nodes show higher strength values than L_M1 in the Task+AgonistStim network, although L_SMA still shows the highest value (Figure 2). Significant statistical differences were found when the strength values of the right cerebellum (*P* < 0.001) or the left thalamus (*P* = 0.006) are compared among the three conditions in 21 subjects by using Friedman test with Bonferroni correction.

**Figure 4:**
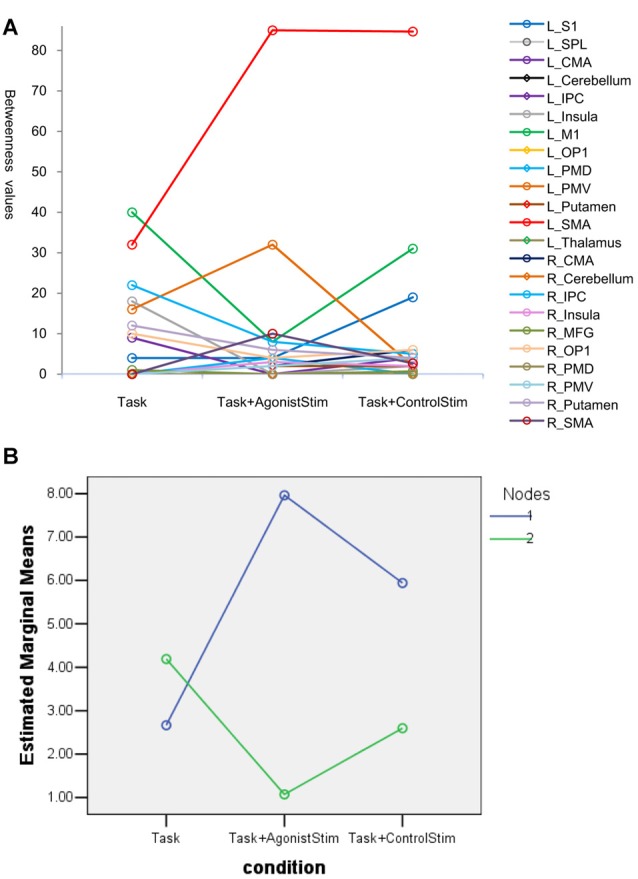
Betweenness values of nodes. **(A)** Many nodes have zero values and are superpositioned on the *x* axis. Topmost nodes in the Task network are L_SMA and L_M1, indicating that these nodes are network hubs. The changes of the two nodes show opposite trends in the Task+AgonistStim network when compared with the Task network, i.e., a higher L_SMA and a lower L_M1 values. Thus, stimulating the agonist muscle remarkably changes the hub of the original network. **(B)** Results of the *post hoc* profile analysis. Blue circles indicate nodes with increased betweenness values in the Task+AgonistStim network when compared with the Task network (Nodes 1). Green circles represent nodes with decreased or unchanged values (Nodes 2). Two groups of nodes demonstrate different trends among three conditions (i.e., unparalleled profiles).

In the Task network, L_SMA and L_M1 show the highest betweenness values, indicating that these nodes are hubs of the network. The two nodes show opposite trends in the Task+AgonistStim network compared with those in the Task network, i.e., a much higher L_SMA (85 vs. 32) and a much lower L_M1 (8 vs. 40, Figure 4A). Thus, stimulating agonists remarkably changes the hub of the baseline (the Task network).

In the *post hoc* profile analysis, we divided 23 nodes into two groups: nodes with increased betweenness values in the Task+AgonistStim network compared with the Task network; and nodes with decreased or unchanged values. The profile analysis can test whether or not each segment of data is the same across all groups. Statistically, the profile analysis is similar to the repeated measure ANOVA. Data pass Mauchly’s test of sphericity with *P* = 0.092. The two groups exhibit different trends under the three conditions (i.e., unparalleled profiles) with *F* = 4.963 and *P* = 0.018 (Figure 4B).

In summary, L_M1 and L_SMA were hubs in the Task network, but L_M1 was not considered a hub in the Task+AgonistStim network.

### Task+AgonistStim Exhibits a Unique Modular Structure

**Figure 5:**
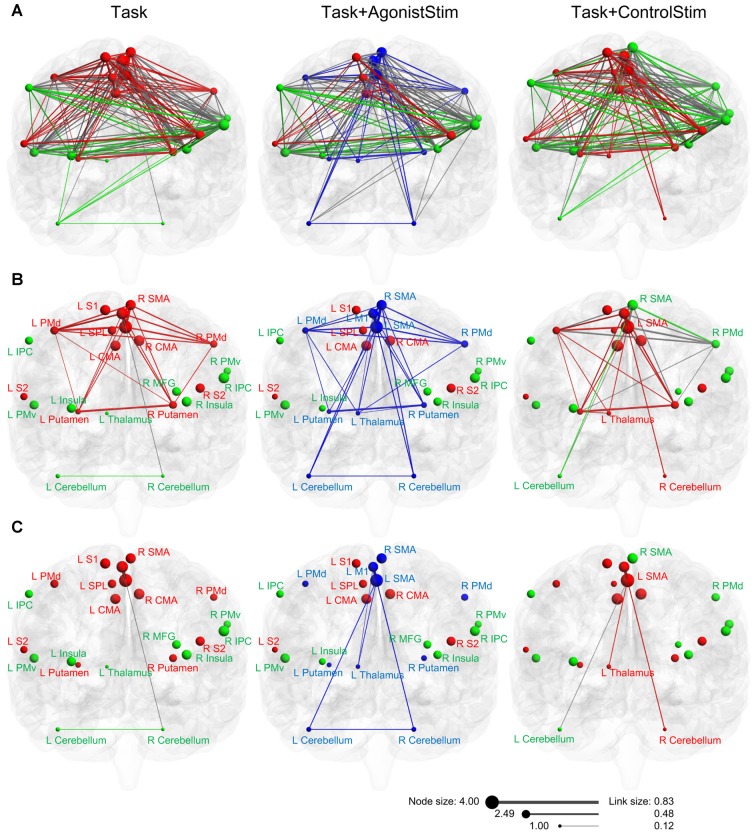
Modules of each network in anatomical space. **(A)** Three networks are visualized with the BrainNet Viewer in the coronal view. Nodes are sized according to nodal strength, and links are sized according to the weight. Nodes in the same module are indicated by the same color. The Task+ControlStim network shows a similar modular structure with the Task network, whereas the Task+AgonistStim network exhibits unique modularity. **(B)** Links among nodes of the new module (blue nodes in the Task+AgonistStim network). Increased numbers of links are observed in three nodes L_thalamus, L_cerebellum and R_cerebellum, especially the thalamus, under conditions receiving sensory stimulation. **(C)** Links between two major centers for cortical motor outputs (L_SMA and L_M1) and three primary sensory relay stations. Only the Task+AgonistStim network shows the link between L_M1 and three primary somatosensory centers (L_Thalamus-L_M1).

Task and Task+ControlStim networks showed similar module structures, whereas the Task+AgonistStim network possessed a unique modular structure (Figure 5A). In this network, the newly appearing module contained three sensory relay stations and major cortical and subcortical motor centers. Conversely, we did not observe high somatosensory centers, including S1, S2, SPL, IPC and the insula.

For each network, we repeated the algorithm 100 times. The modular structure showed 100 identical results.

For Task, Task+AgonistStim and Task+ControlStim networks, the original modular structure and the 100 corresponding results showed low correlation coefficient values of 0.19 ± 0.13, 0.20 ± 0.13 and 0.17 ± 0.12 (mean ± SD), respectively.

### Weak but Critical Links

Fundamentally, the differences under the three conditions are due to somatosensory stimulation. Applied stimulation modulates the network through three primary somatosensory relay stations in the brain, i.e., the thalamus, right cerebellum and left cerebellum.

In the Task network, only one link (R_Cerebellum-L_M1) exists between the sensory relay stations and cortical motor centers. Task+ControlStim and Task+AgonistStim networks contain five and eight of such links, respectively (Figure 5B).

The Task+AgonistStim network contains the highest number of such links; this is consistent with its newly appearing module (due to the highest number of connections between brain regions contained in this module) and its unique network layout (due to the increased number of links connecting three primary sensory centers with other nodes).

Both M1 and SMA are baseline hubs (the Task network). When we focus only on links between these two nodes and three primary sensory relay stations, only one link exists between L_M1 and sensory centers under all conditions (Figure 5C). This link, L_Thalamus-L_M1, exhibits the weakest weight value in the network (beta value 0.12; corresponding correlation value: 0.12). A similar link, namely, L_Thalamus-L_SMA, is also weak in the Task+AgonistStim or Task+ControlStim network (beta value = 0.21, 0.22; corresponding correlation values: 0.21, 0.22). Between each of three sensory relay stations and other nodes, all functional connections are weak links (beta values in three networks range from 0.12 to 0.29; corresponding correlation values: from 0.12 to 0.28).

In the Task+AgonistStim network, the new link L_Thalamus-L_M1 decreases betweenness value of L_M1 by directly sending signals to L_M1 through this new connection during concurrent agonist muscle stimulation. As a result, L_M1 is not required to receive sensory information from multiple sources, including L_IPC, L_Insula, L_S2 and L_Putamen (such links do not exist in the Task+AgonistStim network, Supplementary Figure S3).

## Discussion

In this study, we aimed to see whether simultaneous stimulation of the agonist muscle during voluntary movement induced unique brain activation patterns and network properties compared with movement alone or movement combined with concurrent stimulation over areas without agonist muscles. Activated brain areas observed in this study are also reported in a group of studies on motor function (Garavan et al., 1999; Sahyoun et al., 2004; Dimitrova et al., 2006; Kapreli et al., 2007; Hotz-Boendermaker et al., 2008; Newton et al., 2008; Francis et al., 2009; Trinastic et al., 2010).

### Weak Links Play Critical Roles in Network

The strongest and largest brain activations were evoked by movement combined with stimulation over the agonist muscle. This condition exhibits some unique network features, thereby proving our hypothesis that unique brain mechanisms are induced by movement combined with concurrent somatosensory stimulation over the agonist muscle.

M1 and SMA are major motor output areas in the brain (Gardner and Johnson, 2013; Rizzolatti and Kalaska, 2013; Gallivan and Culham, 2015). SMA plays a general role in contextual control of voluntary movements. Contextual control is the process of selecting and executing actions based on different combinations of internal and external cues. This process also involves withholding inappropriate actions. This role of SMA promotes the creation of a new thalamus–SMA link by stimulation of either the agonist muscle or the control area. M1 converts both central signals on motor intentions and peripheral sensory inputs on the current state of the limb into motor output commands; both S1 and M1 are activated during active movement (Mima et al., 1999). When the agonist muscle is stimulated, the thalamus–M1 link directly strengthens motor outputs via enhanced sensory afferents.

Our findings reveal that concurrent somatosensory stimulation changes the motor network to modulate motor behavior by changing weak but critical links between the three primary sensory centers and the motor cortex, especially the weakest link L_Thalamus-L_M1 in the Task+AgonistStim network. This phenomenon shows that although the three sensory relay stations are nodes with the lowest strengths and betweenness values, these stations play critical roles in the motor control network via weak links.

### Benefits of Thalamus–Motor Cortex Pathway

Several direct fiber connections convey somatosensory inputs from the thalamus to the motor cortex. Somatosensory inputs to the motor cortex mainly arise from S1; the motor cortex including premotor cortex, M1, and SMA also receives several projections from the thalamus (Ghosh et al., 1987). Ninty-three percentage projections from the spinal cord reach the contralateral insula, S2 and CMAs; 4% reaches S1; and only about 3% reaches M1, SMA and other areas (Dum et al., 2009).

To our knowledge, no studies have revealed the function of small amount of projections from the thalamus to the motor cortex. Based on our results, these pathways are “silent” during voluntary movement at normal levels and activated under certain conditions to achieve improved motor control. Hence, primates are equipped with preserved direct links between the thalamus and the motor cortex.

Motor execution needs somatosensory input for feedforward and feedback control; however, sensory feedback from the periphery is noisy (Wolpert et al., 2013; Schoppe et al., 2016). Neural noise can be counterbalanced by slowing down movements when accuracy is needed (Wolpert et al., 2013); when performing precise motor tasks, moving at lower speed is normal for people. Another solution is involuntary or voluntary warm-up actions during preparation for strenuous activities because these actions can activate shortcuts between the thalamus and the motor cortex. Even people without professional sports training rapidly flex and extend their extremities several times before using their limbs to perform heavy or challenging jobs. For athletes, warm-ups involving voluntary movements lead to improved effects than passive stretching alone (McCrary et al., 2015). Hence, motor control is improved when the agonist muscle receives intensive load and the sensory inputs from the muscle to the brain increases.

Long-term training induces adaptations in soft tissues and muscles, and a short duration of warm-up mainly evokes certain neural mechanisms to upgrade motor control. Our findings reveal that increased somatosensory inputs during movement activate links between primary sensory centers (thalamus and cerebellum) and major motor centers (M1, SMA, PMd and PMv; Supplementary Figure S3), i.e., the shortest pathways between brain centers for processing sensory inputs and motor outputs.

These shortcuts provide faster pathways of sensory afferent and allow the motor cortex to acquire original sensory inputs. Thus, the deviation of sensory information is avoided, and the noise is reduced. Hence, the topmost motor output centers, especially M1, use faster, less-inaccurate afferent sensory information to achieve improved feedforward and feedback motor control. This effect is best achieved by stimulating the agonist muscle because the link thalamus-M1 is observed only in Task+AgonistStim. In other words, this condition activates such small amount of projections (≤3%) reported in a previous study (Dum et al., 2009).

### When Weak Links Become Important

It is believed that nodes and links with higher metric values (e.g., weight of links or different measures of node centrality) are more important in networks (Chklovskii et al., 2004; Boccaletti et al., 2006). However, our findings reveal that weak links play pivotal roles in the network.

Strength of weak ties (Granovetter, 1973) is a classic concept in sociology. In a social network, the information flow reaches larger number of people and travels greater distance when passing through weak links rather than strong links. For example, each person is connected to many people. A weak link exists between two persons, A and B, who rarely contact each other; by contrast, strong links exist between A (or B) and his close friends (i.e., people in a same clique/module). When A tells a rumor to his close friends, the message easily spreads within this clique through strong links. However, when A tells the rumor to B (i.e., activating weak links), the message spreads to larger number of people because members of another clique are aware of such rumor.

More efficient information exchanges occur between relatively unfamiliar units in a network through weak ties. Entrepreneurs scored three times higher on a metric of innovation because weak ties expose people to wider range of ideas and non-redundant information (Ruef, 2002). Collaboration between familiar business partners dramatically reduces the probability of investment success, whereas weak ties (ability-matched but unfamiliar partners) enhance investment performance (Paul et al., 2016).

Not all weak links play critical roles. During the stimulation, activated weak links between primary sensory centers and the motor cortex connect two groups that perform distinct functions. These links bridge two heterogeneous groups of brain regions to send unfamiliar (original rather than deviated) sensory information to the motor cortex, thereby remarkably changing the network properties and improving the motor performance. Conversely, low weights at a constant level are noted in the link connecting homogenous brain regions, L_PMd and L_PMv, in the three networks (beta values of 0.19, 0.17 and 0.23).

In biological networks, weak ties are not valued as much as in social networks. We constantly focus on to strong links and consider weak links as trivial. One aspect of networks is reflected by strong links in connecting nodes that perform similar functions, e.g., stronger coupling between left PMd with right PMd (Supplementary Figure S3); such observation agrees with previously reported results (Bestmann et al., 2008). Weak links that bridge heterogeneous brain regions enrich the diversity of information exchanges, maximize information transfer with minimal wiring cost (Gallos et al., 2012), and reflect another aspect of network properties.

### Basic Mechanism of Enhancing Motor Function

We propose that the detected weak but critical link is a basic mechanism for enhancing motor control because it can be used to explain a series of phenomena.

First, humans move their limbs several times before performing strenuous activities. This behavior may be a subconscious way to use these shortcuts and becomes an inherent habit of warming up for immediate improvement of motor performance. Our findings uncover the neural basis on how both subconscious and professional warm-ups prepare muscles for vigorous actions.

Second, many techniques apply various types of somatosensory stimuli to modulate motor behavior in sports training (Wigerstad-Lossing et al., 1988; Willoughby and Simpson, 1998; Lepley et al., 2015) and clinical settings (O’Sullivan, 2000; Paillard, 2008; Kattenstroth et al., 2012). Spatiotemporal summation is believed to be a major neural mechanism of these approaches (Harris, 1984). Nevertheless, motor performance is also improved by activating weak links between the thalamus and the motor cortex, particularly the link thalamus-M1.

Third, similar to external stimulation over muscles, internally enhanced afferent signals from the agonist muscle (i.e., voluntary contraction with high efforts) also activate these weak links and thus enhance motor control. For example, a therapeutic protocol using voluntary articulation and limb movement improves various functions of individuals with Parkinson’s disease (Sapir et al., 2011). This method features intensive effort when performing movement, thereby generating augmented sensory afferents (similar to those resulting from intensive warm-up programs) from highly stimulated agonist muscles.

Finally, several newly-developed medical devices can exert their function through this mechanism; for example, dynamic orthotic clothes for patients with cerebral palsy (Neves, 2013) and tremor-canceling glove for patients with Parkinson’s disease (Mertz, 2016) can generate enhanced proprioceptive inputs and improve motor function. Both devices can use augmented somatosensory afferents for treating diseases with very heavy burdens.

Primates carry preserved weak but critical links to meet the needs for enhanced motor control. Various phenomena and approaches intentionally or unintentionally use this mechanism under both physiological and pathophysiological states. Insights into these weak links and their roles enlighten us to develop and optimize stimulation modalities and parameters for improving the effects of exercise and treatment protocols.

### Limitations and Perspectives

We applied somatosensory stimulation over two different areas to rule out possible effects of confounding factors associated with sensory inputs; these confounding factors may influence network dynamics. In the Task+AgonistStim network, three new connections are formed between major motor output areas (SMA and M1) and the three sensory relay stations when compared with the Task network; in the Task+ControlStim network, the connection between left and right cerebellum disappears and there are fewer number of new connections (Figure 5C). These results indicate that concurrent somatosensory inputs irrelevant to the motor task interfere with the original sensory-motor interaction process.

We focused on investigating brain mechanisms and did not perform additional behavioral measures in this study.

In this work, we aim to reveal mechanisms through which concurrent somatosensory stimulation during movement changes the motor behavior. Our findings reveal that such stimulation changes the brain motor network by modulating weak links between three primary sensory centers and the motor cortex, especially the connection from the thalamus to M1. Scholars reported a small amount of projections from the thalamus to the motor cortex. To the best of our knowledge, this study is the first to explain the functions of these pathways. Our results reveal a basic mechanism of enhancing motor function by activating these weak but critical links. In addition, our findings suggest that the dynamics of networks cannot be understood when considering only strong links and disregarding weak ties.

## Author Contributions

PW, RB and ZL conducted the experiments, PW and BJ analyzed the data and PW designed the experiments. All authors wrote the article.

## Conflict of Interest Statement

The authors declare that the research was conducted in the absence of any commercial or financial relationships that could be construed as a potential conflict of interest.
