# Supplementary material for: Weak but Critical Links between Primary Somatosensory Centers and Motor Cortex during Movement
Source: Front Hum Neurosci. 2018 Jan 17;12:1. doi: 10.3389/fnhum.2018.00001 (PMC5776089; doi:10.3389/fnhum.2018.00001)
Supplement: Supplementary file 1 [file Presentation_1.pdf]

Supplementary Material

Weak but Critical Links between Primary Somatosensory Centers and Motor Cortex during Movement

Pengxu Wei*, Ruixue Bao, Zeping Lv, Bin Jing

*** Correspondence:** Corresponding Author: pengxuwei@gmail.com

1 Supplementary Figures and Tables

1.1 Supplementary Figures

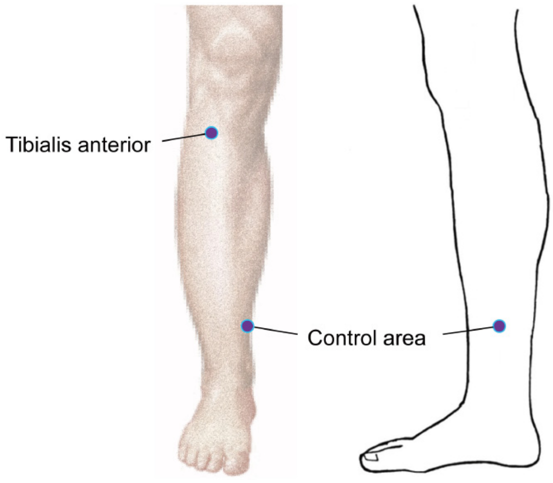

(a)

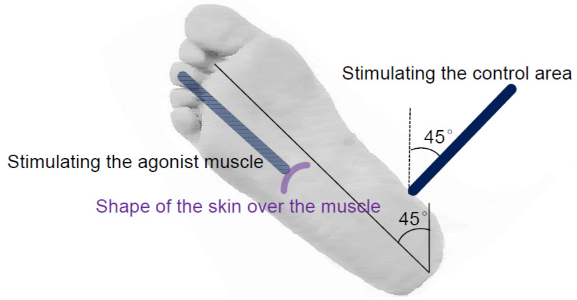

(b)

Supplementary Figure 1. Stimulated areas on leg

(a) Two stimulated areas on right leg. Tibialis anterior is agonist muscle of movement, and control area is located on distal part of medial lower leg. Central point of each area is acupuncture point

selected as stimulation area in a previous study (Bao et al., 2012). We apply manual stimulation by brushing the subject's skin back and forth along the long axis of lower leg using probe with shift distance of 3 cm. (b) Notably, two probes touch the right leg rather than the foot in this perspective illustration to show position of each probe. To stimulate agonist muscle, probe is placed parallel to long axis of foot and thus has same 45° intersection angle relative to vertical line.

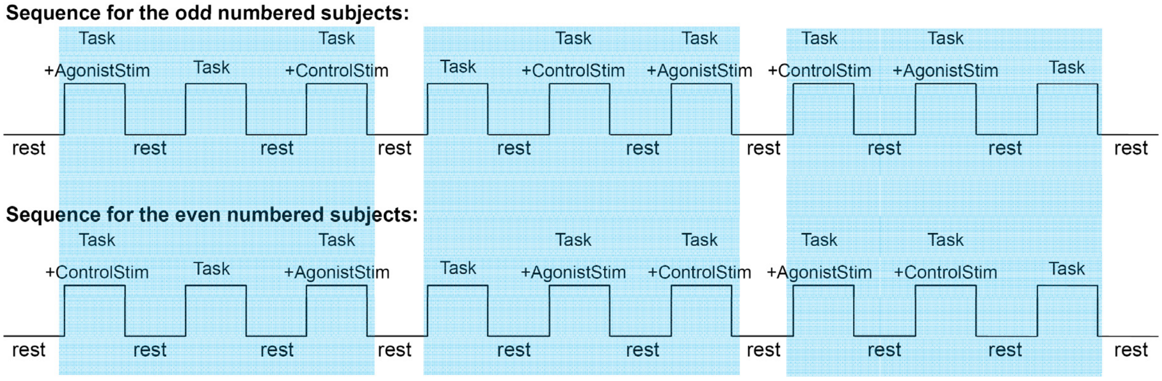

Supplementary Figure 2. Sequence of stimulation. Each blue frame contains three different conditions in different order. Every task or rest period lasts for 30 s.

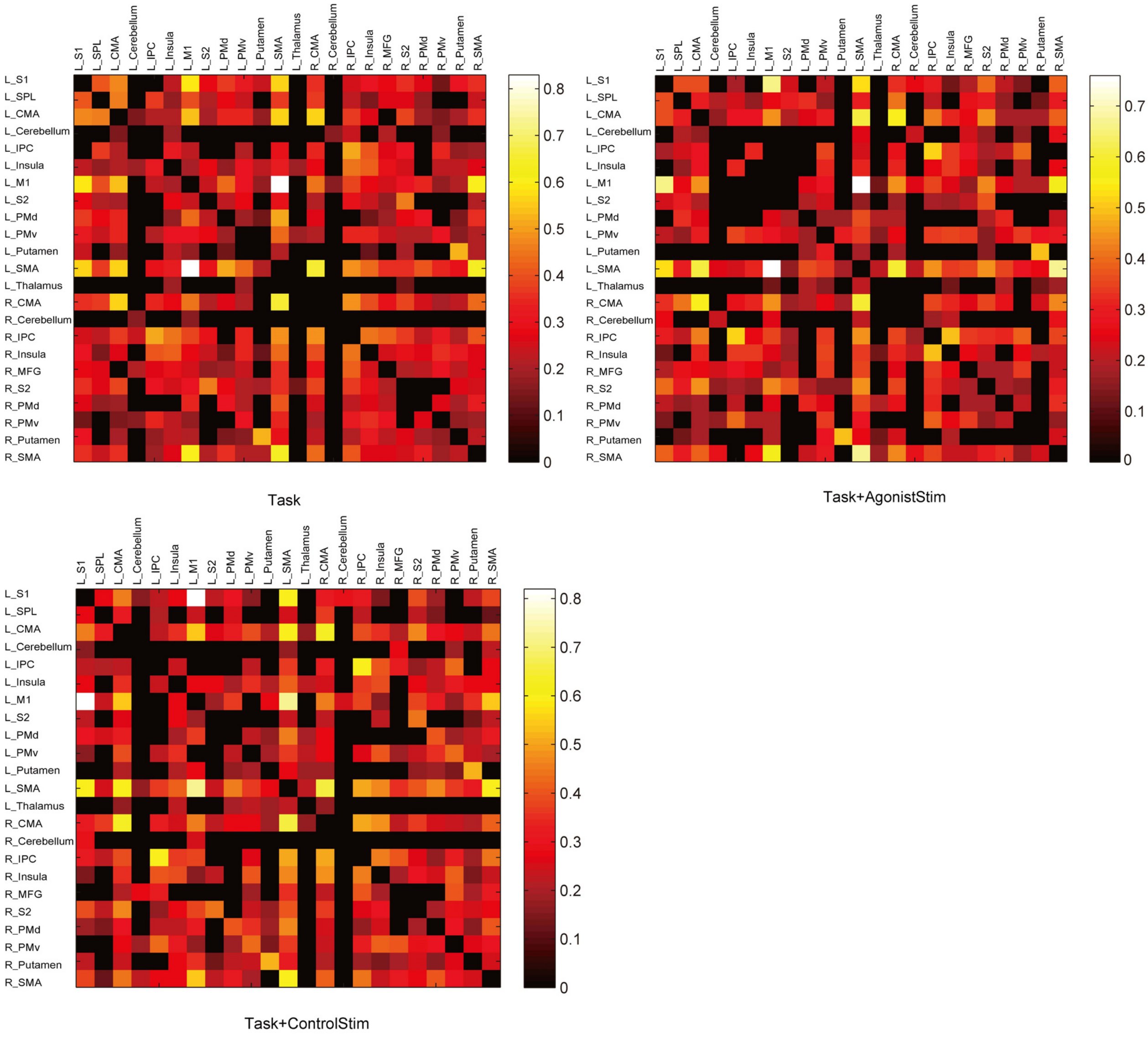

Supplementary Figure 3. Matrix of link magnitudes between all pairs of brain regions. Coloured bars indicate Fisher-transformed correlation coefficient values (beta values). Each grid represents weight value of link between two nodes from corresponding row and column.

1.2 Supplementary Tables

Supplementary Table 1. Locations of node centers.

Centers of nodes	MNI coordinates		
L_SMA	0	-2	56
R_SMA	6	-8	68
L_M1	-2	-28	64
L_CMA	-6	-3	45
R_CMA	8	-1	48
L_PMd	-42	-8	54
R_PMd	52	2	46
L_PMv	-54	4	10
R_PMv	56	12	15
R_MFG	30	46	18
L_S1*	-9	-46	69
L_SPL*	-6	-46	70
L_IPC	-58	-39	34
R_IPC	58	-34	26
L_S2	-52	-22	17
R_S2	44	-30	20
L_Insula	-32	10	8
R_Insula	36	0	12
L_Putamen	-28	-8	6

R_Putamen	28	-4	10
L_Thalamus	-11	-14	5
L_Cerebellum	-40	-58	-32
R_Cerebellum	22	-34	-32

L: left; R: right.

* They are two large brain regions, and centers of mass of their entire scope do not match activated peaks. We also do not find corresponding MNI coordinates in literature. For these two nodes, we thus define a 6 mm sphere with center at [-6 -42 70]; this point is peak location of M1 in a previous study (Francis et al., 2009). We then use intersection between L_S1 (or L_SPL) and 6 mm sphere as scope of node L_S1 (or L_SPL); centers of mass of two scopes are listed in this table.

Supplementary Table 2. Network measures of weighted networks.

**Network measure**	**Description** (Boccaletti et al., 2006)
Degree	The degree of node *i* is the number of edges connected to this node in the network.
Strength	In a weighted network, each edge carries a numerical value that represents the weight of the connection. The strength of a node is the sum of the weights of all the edges connected to this node. It serves as the natural generalization of the degree of node *i*. The strength of a node measures the extent of information transmission between this node and other nodes in the network.
Shortest weighted path length	In a binary network, the shortest path between nodes *i* and *j* is a path with the minimum number of edges between a pair of nodes. In a weighted network, such shortest path is a path between two nodes along which the sum of the weights of its constituent edges is minimized. Hence, the shortest path between two nodes can provide an optimal pathway because a fast transfer is achieved and the use of system resources is minimized.
Weighted characteristic path length	The characteristic path length L is the mean of the geodesic lengths over all pairs of nodes in a network. In a binary network, L of the network is the number of edges in the shortest path between two nodes, as averaged over all pairs of nodes. In a weighted network, the functional distance $D_{i,j}$ between nodes i and j can be defined as $D_{i,j} = 1 - W_{i,j}$, where $W_{i,j}$ is the weight of the edge between nodes i and j.<br><br>The characteristic path length L measures the typical separation between two nodes in a network. For instance, L is the average number of friendships in the shortest chain linking two persons in friendship networks.
Weighted betweenness	The betweenness of a node is the fraction of all the shortest paths in a network that pass through this node and is thus important in controlling information communication across separate parts of the network.
Weighted modularity	Modules are defined as groups of densely interconnected nodes that are only sparsely connected to the rest of the network. Thus, nodes within a module achieve a relatively fast rate of information transmission, and different modules perform different functions with some degree of independence.

Network features were calculated using Brain Connectivity Toolbox (Rubinov,Sporns, 2010) with following functions:

degrees_und.m (degree)

strengths_und.m (strength)

charpath.m (characteristic path length)

betweenness_wei.m (betweenness centrality)

modularity_und.m (modularity)

null_model_und_sign.m (null model generation)

REFERENCES

Bao, R., Wei. P., Li, K., Lu, J., Zhao, C., Wang, Y., Zhang. T. (2012). Within-limb somatotopic organization in human SI and parietal operculum for the leg: An fMRI study. Brain Res *1445*, 30-9.

Boccaletti, S., Latora, V., Moreno, Y., Chavez, M., Hwanga, D.U. (2006). Complex networks: Structure and dynamics. Physics Reports *424*, 175-308.

Francis, S., Lin, X., Aboushoushah, S., White, T.P., Phillips, M., Bowtell, R., Constantinescu, C.S. (2009). fMRI analysis of active, passive and electrically stimulated ankle dorsiflexion. Neuroimage *44*, 469-479.

Rubinov, M., Sporns, O. (2010). Complex network measures of brain connectivity: uses and interpretations. Neuroimage *52*, 1059-1069.
